# Fabrication and Characterization of 2D Nonlinear Structures Based on DAST Nanocrystals and SU-8 Photoresist for Terahertz Application

**DOI:** 10.3390/mi15020203

**Published:** 2024-01-29

**Authors:** Tamara Pogosian, Isabelle Ledoux-Rak, Igor Denisyuk, Maria Fokina, Ngoc Diep Lai

**Affiliations:** 1 LuMIn, ENS Paris-Saclay, CentraleSupélec, CNRS, Université Paris-Saclay, 91190 Gif-sur-Yvette, France; edo-chan@mail.ru (T.P.); isabelle.ledoux@ens-paris-saclay.fr (I.L.-R.); 2 ITMO University, St. Petersburg 197101, Russia; denisiuk@bk.ru (I.D.); mfokina@niuitmo.ru (M.F.)

**Keywords:** nonlinear optics, DAST, nanocrystals, nonlinear grating, QPM

## Abstract

We demonstrate a method for the realization of highly nonlinear optical 4-(4-dimethylaminostyryl)- 1-methylpyridinium tosylate (DAST) two-dimensional structures by a double-step technique. The desired polymeric structures were first fabricated by using the multiple exposure of the two-beam interference technique, and the DAST nanoscrystals were then prepared inside the air-voids of these photoresist templates, resulting in nonlinear periodic structures. The nonlinear properties were characterized by optical and scanning microscopies, as well as by second-harmonic generation technique. This nonlinear modulation is very promising for the enhancement of nonlinear conversion rates, such as terahertz generation, by using the quasi-phase matching technique.

## 1. Introduction

Within the last ten years, the field of terahertz (THz) science and technology has developed greatly. Many new and promising advances in the technology for the generation, manipulation, and detection of THz radiation have been made [1,2]. The most important task is the development of the new materials and technology for THz generation and detection [3,4,5,6,7]. Optical rectification in electro-optical crystals has been demonstrated as the best technique for generating subpicosecond, THz bandwidth radiation [8,9,10,11,12,13]. For THz application, the three most important requirements for nonlinear optical crystals are: (i) high nonlinear coefficient, (ii) low absorption of THz irradiation by the crystal, and (iii) phase matching between pump and THz waves propagations.

Several groups in the world have examined different nonlinear materials for THz emission by optical rectification. These materials include organic salts such as dimethyl amino 4-N-methylstilbazolium tosylate (DAST) [14,15,16,17,18], dielectric inorganic electro-optical materials like LiTaO_3_, and semiconductors such as GaAs and GaP [19]. Among them, DAST is the most efficient THz rectification material [20,21], due to its large electro-optics coefficient and its low dielectric constant (ϵ=5.2), giving rise to a high modulator figure of merit [21]. Indeed, according to data in [22], DAST exhibits a huge second-order nonlinear optical susceptibility $\chi^{\left(2\right)}\;=\;2020\;\pm 220$ pm/V at λ=1318 nm, with its corresponding electro-optical figure of merit being $n_{1}^{3}r_{11}\;=\;\left(530\pm 60\right)$ pm/V at λ=1313 nm. Additionally, it was also demonstrated that DAST possesses the highest damage threshold at a high repetition rate of the pump laser. This is crucial for THz generation, since the highest efficiencies are achieved with maximum peak intensity and optimized central wavelength [16]. However, phase matching between optical wave and THz radiation propagation, a major factor determining the efficiency and the bandwidth of THz emission from a nonlinear crystal, remains as a challenge. Actually, only a few electro-optical crystals possessing a long coherence length are suitable for THz generation. Most nonlinear materials possess a coherence length in a range of few micrometers to dozens of micrometers, thus limiting the THz generation efficiency. It is necessary to find out a way to solve the problem of phase mismatch in DAST crystals.

Quasi-phase matching (QPM) is a technique that compensates the phase mismatch of nonlinear processes by creating a grating of spatially modulated nonlinear properties with a period corresponding to twice of coherence length. Typically, the QPM structures have periods in the range of a few micrometers to dozens of micrometers, which is quite easy to realize today. In a quasi-phase matched structure, the interaction length between THz and pump pulses can be dramatically extended to the centimeters range. For example, QPM structures were used for the generation of THz wave packets with tunable central frequency [23] or for the generation of multicycle narrow-bandwidth THz radiation based on phase synchronous optical rectification [24]. It is clear that nonlinear gratings or QPM structures may provide an efficient control of THz generation.

Conventionally, the QPM effect is obtained in a nonlinear material in which the nonlinear property is modulated between negative and positive signs (+/− QPM). However, this is not easy to realize in practice, in particular for three-dimensional (3D) QPM structures. An alternative way to achieve the same effect is to combine the nonlinear material with a linear material, corresponding to a periodical modulation of $\chi^{\left(2\right)}$ between zero and non-null value (+/0 QPM). The theoretical model of this particular QPM structure was presented in our recent work [25] for a general nonlinear optical effect. Very few works have been demonstrated to realize these +/0 QPM structures. For example, one of our old works [26] demonstrated a layer-by-layer direct laser writing technique to obtain a polymeric 3D QPM structure. This technique has been applied for a Disperse Red 1- poly-methyl-methacrylate (DR1-MMA) side-chain copolymer nonlinear material requiring an additional poling technique, since the copolymer is centrosymmetric. It could be applied for patterning DAST nanocrystals as well, without needing the poling process since DAST nanoscrystal is a naturally noncentrosymmetric material. However, this technique requires the spin-coating of different materials layer-by-layer and thus very time consuming, and imprecise in term of periodicity. Recently, two other groups experimentally demonstrated a direct fabrication by employing a femto-second laser-based bleaching technique [27,28] in LiNbO_3_ material. We have also applied this direct laser writing technique to patterning DAST nanoscrystals structures, as shown in reference [29]. This direct technique allows us to solve the problem of imprecision, but again, it does not produce large QPM structures in a short time.

There are different methods to realize QPM structures based on DAST nonlinear material. However, DAST requires a customized approach, because DAST material is usually fabricated in the form of micro- and nanoparticles. By using the multiple-layer assembly technique, DAST/SiO_2_ multilayers have been proposed for THz generation [30]. We can see that QPM elements based on DAST and SiO_2_ micrometer-size layers can be effective for THz generation, but their fabrication is still a great challenge. In our recent work, we have demonstrated a method to obtain the desired DAST structures by mixing DAST nanocrystals with a passive polymer matrix. Indeed, we have successfully obtained a nonlinear nanocomposite based on DAST nanocrystals embedded in a PMMA polymer matrix and showed that the nonlinear conversion efficiency does not depend on the DAST crystals size [31]. We have also fabricated 2D grating structures by photobleaching DAST nanocrystals using a technique called one-photon absorption-based direct laser writing [29]. The last one is very efficient but rather slow and therefore time-consuming. Very recently, we proposed an alternative method by combining a mask lithography and thermal annealing to produce a +/0 QPM DAST structure [32]. Highly efficient THz generation by this DAST nanocomposite is demonstrated upon excitation by femtosecond pulses and generation by the method of optical rectification. However, since the mask technique was used, it is not suitable to adjust the structures configurations and periodicity, and also it is not possible to produce small periods and 3D structures. Therefore, it is necessary to look for a method that should be rapid, and allows the production of multi-dimensional and large QPM structures.

**Figure 1:**
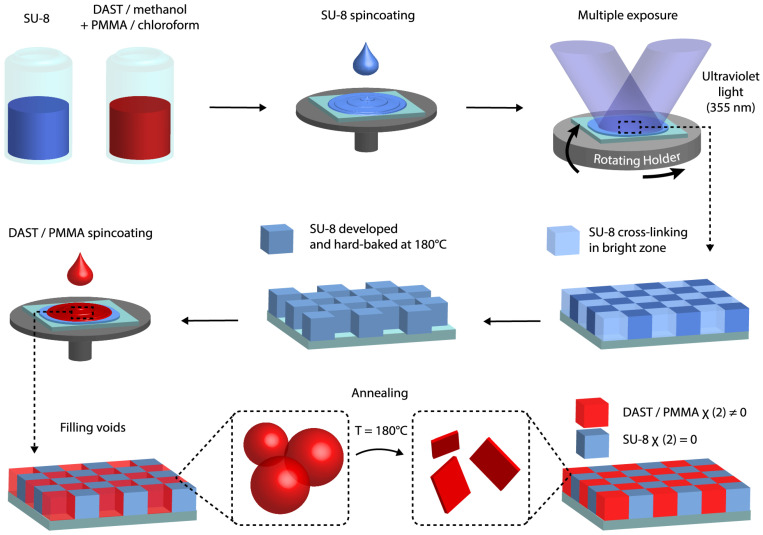
Fabrication process of +/0 QPM structure by filling voids of a photoresist template with DAST nonlinear material. The template is fabricated by the multiple-exposure two-beam interference technique. The DAST nanocrystals are obtained by the thermal annealing method.

In this work, we demonstrated an alternative way to fabricate DAST/polymer large structures in a short time, which is based on filling a holographically fabricated SU-8 template by a DAST-PMMA composite. Figure 1 shows the fabrication process of desired DAST/polymer structures. First, this method consists in the preparation of the SU-8 template by an interference technique [33], which has many advantages such as rapidity, simplicity, and large, uniform structures. Second, the DAST-PMMA composite is spin-coated into SU-8 template and thermally annealed to remove the solvent and to induce DAST crystallization. The final result is a linear/nonlinear structure with a controllable period and desirable configurations, which is equivalent to a +/0 QPM structure, and which could be useful for THz generation. We demonstrated the nonlinear properties of these fabricated structures by measuring the second-harmonic generation (SHG) signals.

## 2. Theory and Numerical Calculations of Two-Beam Interference Technique

### 2.1. Theory of Two-Beam Interference

The fabrication of one-, two- and three-dimensional (1D, 2D, and 3D) micro- and nanostructures is of great interest nowadays. The multi-beam interference technique allows different photoresist-based structures to be obtained depending on the number of laser beams and their arrangement. The common advantage for all holographic techniques is a parallel fabrication process, which results in uniform structures over a large area and in a short fabrication time. However since multiple laser beams should be used, the fabrication setup becomes quite complicated [34,35]. The two-beam interference technique processes many advantages because of its simplicity [33]. In particular, this technique provides high contrast between the minimal and maximal intensities of interference pattern because of the identical polarization of two laser beams in the interference area. Furthermore, the multi-exposure of the two-beam interference pattern allows the realization of various structures with different configurations in multi-dimensions. In this work, we employed this two-beam interference technique to realize DAST nonlinear photonic structures.

**Figure 2:**
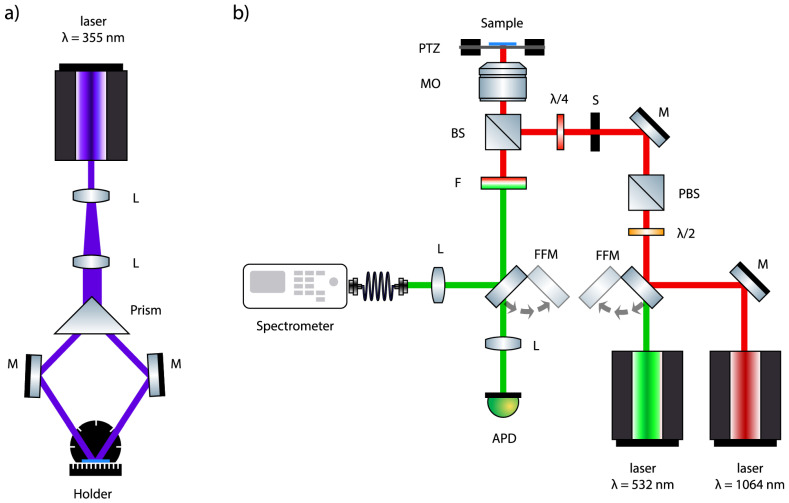
(**a**) Two-beam interference setup used for the fabrication of multidimensional polymeric templates. L: lenses; M: mirrors. The sample is placed in a holder that can rotate in two dimensions for multiple exposures; (**b**) confocal laser scanning setup used for the characterization of QPM structures. PZT: piezoelectric translator; MO: microscope objective; λ/4: quarter-wave plate; λ/2: half-wave plate; BS: beam splitter; PBS: polarizing beam splitter; M: mirror; FFM: flip-flop mirror; S: electronic shutter; L: lens; F: infrared filter; APD: avalanche photodiode.

Figure 2a illustrates the schematic setup of the two-beam interference technique. A lenses system was used to enlarge the initial laser beam and to obtain a large and uniform working area. The extended beam is divided by a prism into two beams of the same profiles and intensities, which are then deviated by two reflecting mirrors towards the photoresist where they interfere, resulting in an intensity modulation. The electric fields of two laser beams can be written as:

(1) E1(r,t)=Re[E01ei(k1.r−ωt)],

(2) E2(r,t)=Re[E02ei(k2.r−ωt)],

where $k_{1}$ and $k_{2}$ are the wave vectors of two laser beams, **r** is the position vector in the overlapping space, and $E_{01}$ and $E_{02}$ are the amplitudes of electric fields. The sum of the electric field of individual plane waves is modulated (interference) at overlapping areas:

(3) $$E_{T}\left(r,t\right)=E_{1}\left(r,t\right)+E_{2}\left(r,t\right).$$

The interference intensity distribution of the resultant wave is given by

(4) $$I_{T}\left(r,t\right)={\langle E_{T}^{*}\left(r,t\right).E_{T}\left(r,t\right)\rangle }_{t},$$

where ${\langle \ldots \rangle }_{t}$ represents the time average of the resultant electric field. The interference of two laser beams is a 1D pattern, whose period (Λ) is calculated by

(5) $$\Lambda =\frac{\lambda }{2\sin\theta },$$

where θ is a half-angle between two laser beams and λ is the wavelength of the laser beams. In our case, we assume that the polarizations of two beams are the same (vertical direction) for all values of θ-angle allowing to obtain 1D structure with maximal and same contrast for any periodicity. According to the Equation (5), the structure period can be adjusted from as small as λ/2, i.e., few hundreds of nanometers, to as large as we want. It means that this is fully adaptable to realize the QPM structures with desired period, which is twice the coherence length of the nonlinear crystal and usually in the range of micrometers.

**Figure 3:**
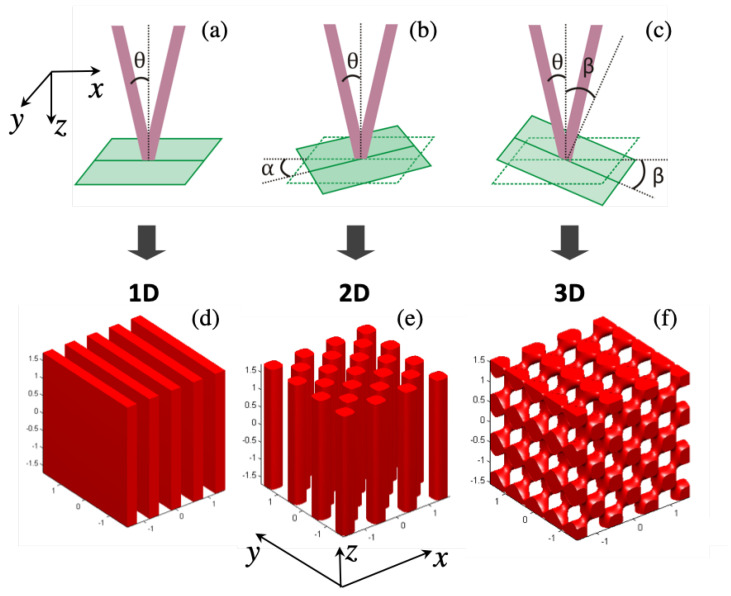
Illustration of θ–angle (**a**), which is half of the angle between two beams, and rotation angles α (**b**) and β (**c**). Matlab simulations of iso-intensity distribution ($I_{iso}=0.6I_{max}$) of two-beam interference with λ=355 nm, $\theta =15^{{}^{\circ}}$: (**d**) one exposure with (α,β) = ($0^{{}^{\circ}},0^{{}^{\circ}}$); (**e**) two exposures with (α,β) = ($-45^{{}^{\circ}},0^{{}^{\circ}}$) and ($45^{{}^{\circ}},0^{{}^{\circ}}$); (**f**) three exposures with (α,β) = ($90^{{}^{\circ}},0^{{}^{\circ}}$), ($0^{{}^{\circ}},45^{{}^{\circ}}$) and ($180^{{}^{\circ}},45^{{}^{\circ}}$), respectively.

As is shown in the Figure 2a and Figure 3, the photoresist sample is fixed on a rotating holder. Both rotation angles, α and β, influence resultant 1D structure. Therefore, we realize a rotation of the 1D interference pattern around two axes, *z* by an angle α, and *y* by an angle β, as seen in Figure 3b,c. The corresponding rotation matrices are:

(6) $$R_{z}\left(\alpha \right)=\left[\begin{array}{ccc} \cos\alpha & -\sin\alpha & 0\\ \sin\alpha & \cos\alpha & 0\\ 0 & 0 & 1 \end{array}\right],$$

(7) $$R_{y}\left(\beta \right)=\left[\begin{array}{ccc} \cos\beta & 0 & \sin\beta \\ 0 & 1 & 0\\ -\sin\beta & 0 & \cos\beta \end{array}\right].$$

In this case, the resultant intensity distribution becomes:

(8) $$I_{\alpha ,\beta }=2E_{0}^{2}\cos^{2}\left[kz\sin\theta \sin\beta +k\cos\theta \cos\beta \left(x\cos\alpha +y\sin\alpha \right)\right],$$

where |$E_{01}$| = |$E_{02}$| = $E_{0}$.

If a multiple-exposure of the two-beam interference pattern is applied, the total intensity from all exposures is calculated by:

(9) $$I_{\mathrm{multiple}}=\sum_{i}I_{\alpha_{i},\beta_{i}},$$

where i=1,2,3,… represent the number of exposures realized at corresponding $\alpha_{i}$ and $\beta_{i}$ angles.

### 2.2. Numerical Calculations

By using personal MATLAB codes, we simulated the resultant structures for different exposures with different parameters. If α and β are equal to zero and only one exposure is applied, it is possible to obtain a simple 1D structure as shown in Figure 3d. Additional exposures with β=0 and α≠0 provide 2D structure as shown in Figure 3e. The configuration of 2D structures depends on the number of exposures and the rotation α–angle. For example, with only two exposures, a 2D square structure is obtained with $\alpha =0^{{}^{\circ}}$ and $\alpha =90^{{}^{\circ}}$ and a 2D hexagonal structure is obtained with $\alpha =0^{{}^{\circ}}$ and $\alpha =60^{{}^{\circ}}$. Moreover, 2D quasi-periodic structures can be also created in this way with more than three exposures. One more rotation by a β–angle leads to fabrication of 3D structures, as shown in Figure 3f. Here, again, the two-beam interference technique with multiple-exposure allows any 3D structure to be obtained on demand, depending on the number of exposures and the rotation α and β angles.

**Figure 4:**
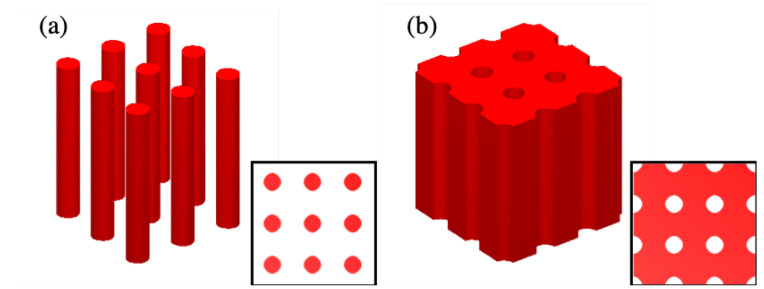
Matlab simulations of iso-intensity distribution of two-beam interference with λ=355 nm, $\theta =15^{{}^{\circ}}$ and with two exposures with (α,β) = ($0^{{}^{\circ}},0^{{}^{\circ}}$) and ($90^{{}^{\circ}},0^{{}^{\circ}}$), respectively. (**a**) “Cylinders” structure obtained with an $I_{iso}=0.8I_{max}$. (**b**) “Air-holes” structure obtained with an $I_{iso}=0.2I_{max}$.

One of the advantages of the interference technique is that it allows the filling factor of fabricated structures to be controlled. Indeed, manipulations with exposure time or power of the laser beam give a precise doses control. It leads to a possibility to fabricate a variety of structures from so-called “cylinders” (Figure 4a) to “air holes” (Figure 4b). The longer exposure time, the more photoresist becomes cross-linked (in the case of negative SU-8 photoresist), resulting in the “air holes” structure. In contrast, short exposure time or less power results in separate cylinders. Controlling the filling factor is thus useful to obtain better efficiency for different QPM orders [25].

In this work, we will demonstrate the idea by simply fabricating SU-8 polymeric 2D periodic structures, filling their air-voids with DAST-PMMA nanocrystals and characterizing their SHG signal.

## 3. Experimental Section

### 3.1. Preparation of Polymeric Template

SU-8 2002 (Micro. Chem. Corp., Newton, MA, USA) was spin-coated onto glass substrates with a thickness of 2 μm. The sample was soft-baked at 65 °C for 1 min and then at 95 °C for 2 min to remove the residual solvent. For the preparation of multi-dimensional (1D, 2D, and 3D) periodic structures, we have used a multiple-exposure two-beam interference method. For that, a continuous-wave (cw) UV laser (Cobalt) with λ=355 nm was used, as shown in Figure 2a. After light exposure, the sample was post-baked on a hot plate at 65 °C for 1 min and then at 95 °C for 5 min. The sample was then developed by a SU-8 developer for 4 min, and rinsed by isopropanol and DI water. Finally, the sample was hard-baked for 40 min at 180 °C. This annealing of SU-8 matrix at high temperature is needed to remove residual solvents for the proper environment for further DAST crystallization.

### 3.2. Preparation of DAST/PMMA Solution

PMMA (Plexiglas^®^ V045, Trinseo, Courbevoie, France) was dissolved in chloroform (CL0218 Scharlau, Barcelona, Spain) and DAST (CAS:80969-52-4 Genolite biotek, Portland, OR, USA) was dissolved in methanol (322415 Aldrich, St. Louis, MO, USA). The DAST mass is equal to 2% of the PMMA mass, and DAST + PMMA is 15% of mass in chloroform. The amount of methanol was calculated from the solubility limit of DAST in methanol at room temperature [36]. Dissolved DAST was added into PMMA solution and was stirred together to achieve a DAST/PMMA solution with high homogeneity.

### 3.3. Mechanism of Crystallization

The formation of nanocrystals in PMMA is based on DAST aggregation, upon which the size of particles is limited by the diffusion of material through the viscous polymer solution. Both solvents used for PMMA and DAST have close boiling temperatures and relatively close vapor pressures. At the beginning of the process, PMMA and DAST are dissolved in the mixture of two solvents. The formation of the PMMA matrix and DAST nanocrystals in the mixture starts with centrifugation and terminates after complete evaporation of the solvent during annealing. First, the DAST/PMMA composite was dropped on the SU-8 template and spin-coated at a low speed to remove extra solution from the top. It forms amorphous DAST particles distributed in PMMA. Second, during annealing at an elevated temperature of 180 °C for 30 min, amorphous particles transform into a noncentrosymmetric crystalline structure. The mechanism of the formation of DAST crystals in the PMMA matrix and the effect of the synthesis parameters on the crystallite size were considered in detail in our previous works [29,31,37].

It is important to note that the nonlinear property of the DAST nanoparticles remains the same for any particle size. But due to the fabrication of DAST crystal in SU8 matrix of 2 μm thickness, it is not possible to use a transmission electron microscope to investigate the influence of polymeric structures on DAST nanoparticles’ size and density. However, thanks to the previous work [31], it is evident that the DAST nanoparticle size is less than 200 nm for a mass concentration of 2%, while the air-voids size of the SU-8 structure is about 1.5 μm (half of the structure period); we believe that this does not significantly affect the formation of DAST nanocrystals and thus their size and density.

### 3.4. Sample Characterization

The linear and nonlinear properties of fabricated samples were characterized by different optical instruments, such as optical microscope, electron scanning microscope, as well as confocal laser scanning microscope. Figure 2b shows a laser system used to characterize both linear and nonlinear properties of DAST periodic structures. Indeed, DAST crystals can absorb and emit light as a fluorescence emitter. For that, the sample was excited by a cw 532-nm Oxxius frequency-doubled Nd-YAG laser (maximum power: 300 mW, coherence length: 300 m; pointing stability: 0.005 mrad/C), and the fluorescence signal was collected by the same confocal system. DAST crystals also possesses a strong nonlinear response, as mentioned above. In our case, the SHG signal of DAST nanocrystals was demonstrated by exciting the sample by a pulsed 1064 nm laser (maximum averaged power: 80 mW; pulse duration: 1 ns, repetition rate: 24.5 kHz). These two lasers, 532 nm and 1064 nm, are perfectly aligned in front of the objective lens (OL), and were used alternatively, depending on the desired measurement. The laser beam is focused into the sample by a high numerical aperture OL. The fluorescent or SH signals were detected by the OL and sent back to the avalanche photodiode (APD), after filtering out the excitation light beam. By scanning the focusing beam (movement of the piezoelectric translator (PTZ)), we can obtain a color map of fluorescent or SHG signals, which confirm the creation of periodic linear/nonlinear patterns. The emission spectra, fluorescence or SHG, were also analyzed by using a spectrometer.

## 4. Results and Discussion

**Figure 5:**
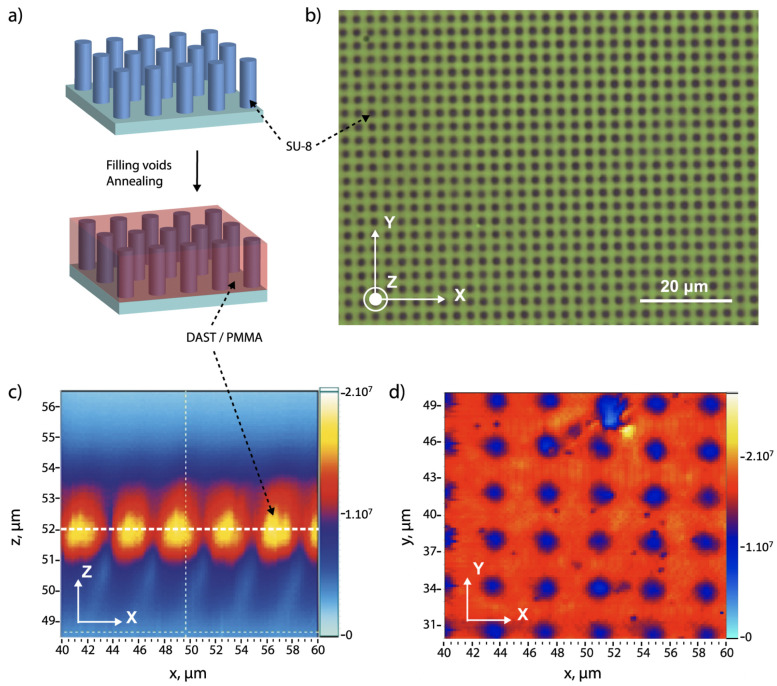
(**a**) Simulationof the photoresist structure. (**b**) Optical microscope image of SU-8 2D square structure fabricated by two-beam interference method. The black/grey color represents the SU-8 pillars. (**c**,**d**) Fluorescence mapping images of fabricated sample by using a cw 532 nm laser beam. (**c**) Scanning along zx-plane. (**d**) Scanning along xy-plane at a *z*-position as marked by the white dashed line of the image shown in (**c**).

To demonstrate the idea of our proposal, we fabricated different 2D SU-8 templates by using the multiple-exposure two-beam interference technique with a period of 3 μm. The fabrication of the 2D structure depends on the number of exposures and on sample orientation with respect to each exposure [33]. For example, two mutually perpendicular positions of the sample allowed us to fabricate 2D square structures, as shown in Figure 5b. Furthermore, different types of structures, such as air-voids or polymer-cylinders as shown in Figure 4, both having circular shapes, could be fabricated on demand. Indeed, we have demonstrated that by controlling the exposure dose, i.e., exposure time or laser power, the polymerization rate can be different, resulting in 2D structures with material cylinders (low dose) or air-holes (high dose). For the same laser power, the exposure time used to obtain air-hole structures was generally two times longer than that used to produce material cylinders structures. We also obtained, for example, a square-shaped structure consisting of air-voids or material cylinders when the dose is in between low (for material cylinder structures) and high (for air-voids structures) doses. Figure 5a only illustrates the case with the low-exposure dose, i.e., material cylinder structures. Thus, by using a modest exposure dose (2.1 mW/cm^2^ per interference beam and 2 s for each exposure), this fabricated structure is made of SU-8 pillars, as shown in Figure 5a,b. We note that this experimental result presented in this section is different to the schematic one presented in Figure 1, but this does not influence the fabrication mechanism and the physics of the QPM technique based on fabricated structures.

**Figure 6:**
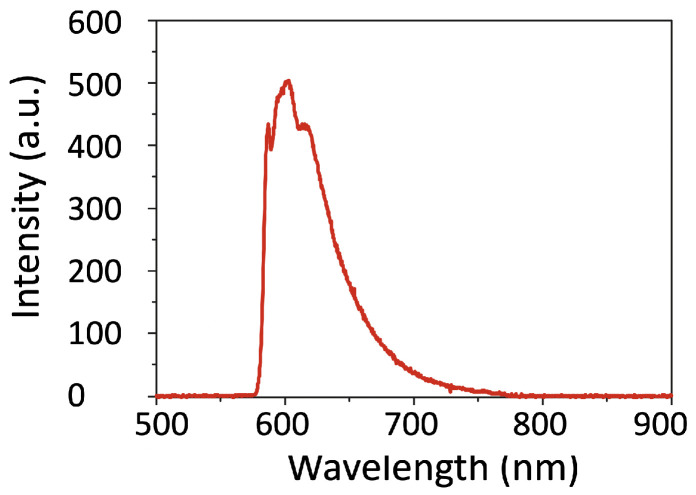
Fluorescence spectra of DAST nanocrystals in 2D structure, obtained by exciting at 532 nm wavelength. In this experiment, a filter with a cut-off wavelength of 580 nm was used.

Figure 5c,d shows the fluorescence mapping image of the final sample, after filling the air-areas of the SU-8 template by the DAST nanocrystals. It is well seen that SU-8 pillars with a weak fluorescence signal are surrounded by DAST/PMMA composite, where DAST crystals show a fluorescence quantum yield of 14–20% [38] and provide a strong fluorescence signal. By scanning the sample along transverse and longitudinal directions, we obtain complete information, i.e., the structure thickness and periodicity. The sample thickness agrees well with the value measured by a profilometer, and the structure period is also very consistent with the designed structure. The fluorescence spectrum of DAST/PMMA area (Figure 6) shows typical fingerprint of DAST crystals with noncentrosymmetric “red-green” form [37,39].

**Figure 7:**
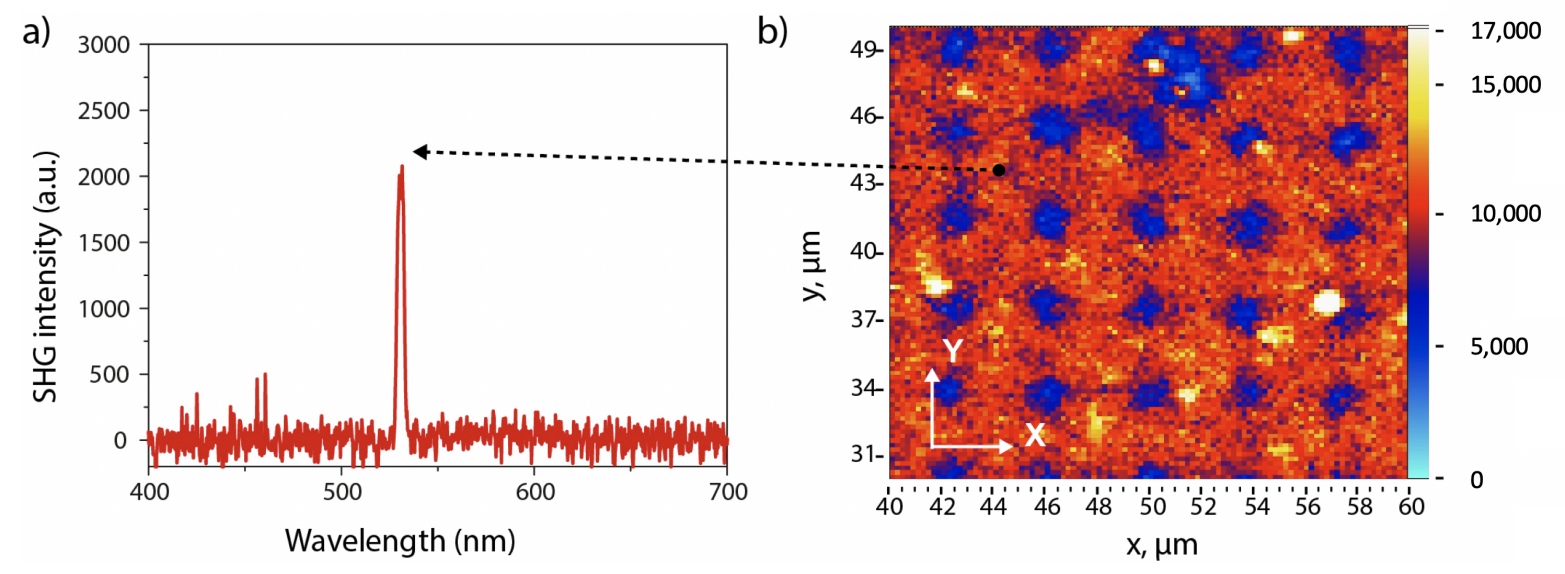
SHG of DAST nanoparticles embedded in a 2D periodic structure, obtained by using a fundamental laser beam at 1064 nm. (**a**) SHG spectrum. (**b**) SH mapping image obtained by scanning the sample in xy-plane.

Similar to the fluorescence experiment, when using a 1064 nm pulsed laser, we obtained the SHG mapping image of corresponding sample. Figure 7 shows the SHG wavelength and mapping image. A clear peak at 532 nm was obtained as a proof of the nonlinear property (SHG) of the DAST nanocrystals. Figure 7b shows that the SH signal is very high from DAST nanocrystals, as compared to the weak signal from SU-8 pillars. Thus, a periodic linear/nonlinear microstructure has been demonstrated by this scanning method. The advantage of this structure as compared to those fabricated by other methods is that there exists a clear border between linear (SU-8 photoresist) and nonlinear (DAST nanocrystal) responses, and the structure is also very large, since it was realized by the interference method, for which the structure area depends on the diameter of the interference laser beam. Unfortunately, we cannot realize QPM measurements with these samples since the film thickness is only 2 μm. However, this work demonstrates that the combination of interference template and thermal annealing method is an excellent way to obtain rapidly large QPM structures. We expect to investigate this method for 3D QPM, and apply it for efficient THz generation.

It is important to discuss the fabrication of 3D structures for QPM application. Indeed, for the QPM effect, the periodicity is in the range of few μm, or even longer for THz generation. Also, to be efficient, the number of layers should as high as possible. This means that the thickness of the 3D QPM structures should be in the range of millimeters or centimeters. However, due to the absorption of the photoresist, it is not possible to obtain a 2D or 3D structure with such thickness. This is because the photoresist’s absorption reduces the laser intensity resulting in a modification of the structure throughout the film thickness. With the one-photon absorption method, the thickness of fabricated structures is limited to only few μm. To obtain thick 2D and 3D structures, a method based on two-photon absorption should be used. This method is well-known in case of direct laser writing, as explained above [27,28,29]. However, this two-photon absorption based method is still not evident in case of two-beam or multiple-beam interference due to the requirement of using a high-power laser, such as femto-second laser [34,40,41]. We recently developed an alternative idea by using the one-photon absorption-based technique to increase the film thickness of the interference pattern to 100 μm [42]. The technique consists of using low absorption material and exposing the sample once again by an additional and independent counterpropagating uniform beam, which allows compensation for the diminution of the light intensity of the interference pattern. In the very near future, we expect to be able to realize such a 3D QPM structure for DAST crystals, and in particular the QPM suitable for THz generation.

## 5. Conclusions

In this work, we demonstrated a novel method to realize large nonlinear QPM structures for efficient nonlinear conversion. The proposed method is based first on SU-8 template preparation by a conventional interference method. Then, a PMMA-DAST solution was deposited by spin coating and DAST nanocrystals were formed in the air voids of SU-8 template via the thermal annealing method. The nonlinear structure was characterized by SHG and fluorescence 3D microscopy. Mapping results evidence the formation of the red crystalline form of DAST nanocrystals grown in the holes of the SU-8 matrix, forming well-delimited periodic nonlinear domains, which opens interesting perspective for QPM-based nonlinear applications of these structures, such as THz emission by optical rectification.

## Data Availability

Data are contained within the article.
